# Novel Adiposity and Biochemical–Anthropometric Indices to Identify Cardiometabolic Risk and Metabolic Syndrome in Mexican Adults

**DOI:** 10.3390/healthcare9111561

**Published:** 2021-11-16

**Authors:** Patricia Lizett Rodríguez-Carrillo, Priscila Irene Aguirre-Tostado, Maciste H. Macías-Cervantes, Jorge Alejandro Alegría-Torres, Claudia Luevano-Contreras

**Affiliations:** 1 Department of Medical Sciences, University of Guanajuato, León 37320, Mexico; pl.rodriguezcarrillo@ugto.mx (P.L.R.-C.); pi.aguirretostado@ugto.mx (P.I.A.-T.); mh.macias@ugto.mx (M.H.M.-C.); 2 Department of Pharmacy, University of Guanajuato, Guanajuato 36000, Mexico; ja.alegriatorres@ugto.mx

**Keywords:** visceral adipose tissue, cardiometabolic risk, metabolic syndrome, adiposity indices, insulin resistance indices

## Abstract

Although several indices used in clinical practice identify cardiometabolic risk (CR) and metabolic syndrome (MetS), it is imperative to develop indices for specific populations. Therefore, we proposed and validated sex-specific indices to identify CR associated with visceral adipose tissue (VAT) accumulation or MetS in Mexican adults. Additionally, a cut-off value for the visceral fat area (VFA) to identify CR was proposed. Clinical, anthropometric, biochemical, and body composition variables were evaluated in 904 subjects (25–45 years old) (84.4% men). Multiple and logistic regressions were used to model the indices and ROC curve analysis to determine predictive performance. An additional cohort (*n* = 186) was used for indices validation, and Cohen’s kappa coefficient was employed for agreement analysis. The proposed sex-specific indices, called Mexican adiposity indices (MAIs) and biochemical–anthropometric indices (BAIs), were good predictors for CR and MetS. The kappa coefficients showed a moderate agreement level. The VFA cut-off value chosen to identify CR was 100.3 cm^2^ because it had the best combination of sensitivity (66.8%) and specificity (64.4%). MAIs and BAIs could be clinical tools to identify either CR associated to VAT accumulation or MetS, respectively. A VFA cut-off value of 100.3 cm^2^ could identify CR in Mexican men.

## 1. Introduction

Abdominal visceral fat plays a central role in cardiometabolic risk (CR) development [1]. It has been demonstrated that visceral adipose tissue (VAT) has a strong association with insulin resistance (IR), metabolic syndrome (MetS) incidence, and the consequent development of cardiovascular disease, diabetes, and higher mortality rate [2,3,4].

The estimation of visceral fat area (VFA) may be helpful to recognize subjects with CR. Different methods are available to assess VFA; among the most widely used are computational tomography, magnetic resonance imaging, dual-energy X-ray absorptiometry, and bioelectrical impedance analysis (BIA) [5]. In 2002, the Japanese Society for the Study of Obesity (JSSO) proposed a cut-off value for VFA (100 cm^2^) as a surrogate measurement for increased CR [6]. However, this cut-off value is not specific for the Mexican population, and methods to estimate VFA are expensive, and their use in daily clinical practice is limited.

Anthropometric measurements, such as body mass index (BMI), waist circumference (WC), and waist-to-height ratio (WHtR), are moderate predictors of individual CR factors in Mexican children and young adults (16–22 years) [7,8,9]. WHtR could predict hypertriglyceridemia, whereas WC could predict hyperglycemia [8]. Another study demonstrated that WC was only a moderate predictor of MetS, and BMI was a good predictor of CR only with another anthropometric and biochemical measurement [9]. Therefore, additional indices are needed to identify global CR in Mexican adults.

Hence, adiposity indices (AIs) and insulin resistance indices (IRIs) combining anthropometric and biochemical variables have been proposed as clinical practice tools to identify CR and MetS. Some AIs are the visceral adiposity index (VAI) and the lipid accumulation product (LAP) [10,11]. The most used IRIs are the triglycerides–glucose index (TyG), TyG–waist circumference index (TyG–WC), TyG–body mass index (TyG–BMI), and the metabolic score for insulin resistance (METS-IR) [12,13,14]. However, these AIs and IRIs have limited validity to the population used for their development.

Consequently, some researchers have proposed new population-specific indices with better performance. For instance, the new VAI (NVAI) for the Korean population to predict cardiovascular diseases [15] and the metabolic score for visceral fat (METS-VF) for the Mexican population to predict type 2 diabetes mellitus (T2DM) and hypertension have been proposed [16].

Over the last 30 years, obesity and central obesity prevalence have increased in Mexico [17,18]. Hence, it is imperative to develop specific indices to identify the effect of obesity and VAT accumulation on CR and MetS incidence, and these indices should be easy to use in the clinical setting.

Therefore, the purpose of this study was to propose and validate novel indices to identify CR associated to VAT accumulation and MetS incidence. Furthermore, a cut-off value for VFA assessed by BIA (VFA–BIA) to identify CR in Mexican adults was proposed.

## 2. Materials and Methods

### 2.1. Subjects

A comparative, cross-sectional study was conducted in men and women between 20 and 60 years old with a BMI of 18.5–40.0 kg/m^2^. One thousand three hundred and ninety-five subjects completed clinical, anthropometric, body composition, and biochemical assessments at the Department of Medical Science at the University of Guanajuato.

**Figure 1:**
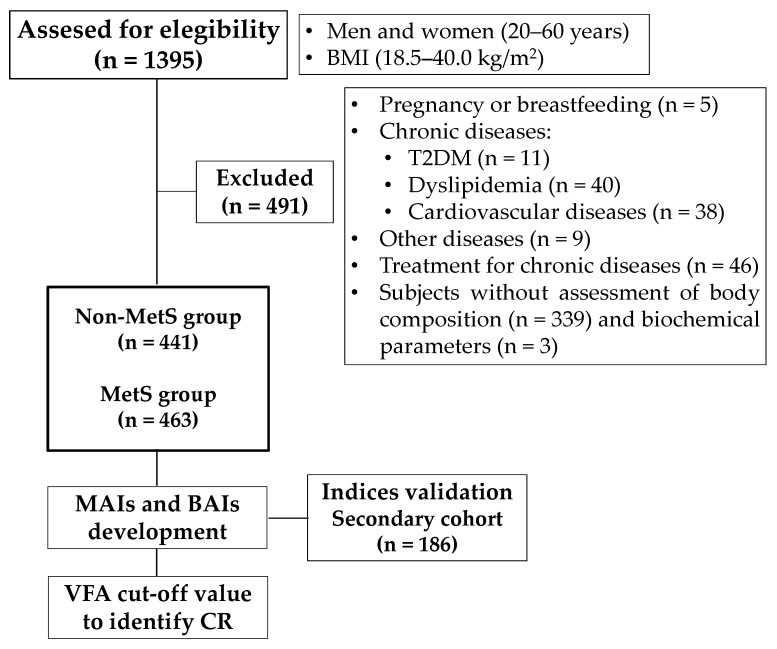
Study design flowchart. BMI: body mass index, T2DM: type 2 diabetes mellitus, MetS: metabolic syndrome, MAI: Mexican adiposity index, BAI: biochemical–anthropometric index, VFA: visceral fat area, and CR: cardiometabolic risk.

Women who were pregnant or breastfeeding were not included, neither were subjects who self-reported chronic diseases: T2DM, dyslipidemia, and cardiovascular diseases (myocardial infarction and atherosclerotic cardiovascular disease) and with the following treatment: hypoglycemic medications, antihypertensive medications, diuretic drugs, lipid-lowering drugs, and anti-obesity medications. Subjects to whom body composition and biochemical parameters could not be measured were not included (Figure 1). For the final analysis, 904 subjects were included in the main cohort.

An additional cohort (secondary cohort) was used for indices validation (*n* = 186). The same selection criteria and assessments were carried out in this cohort.

The study protocol was conducted following the Declaration of Helsinki, and the Ethics Committee of the University of Guanajuato approved it (CIBIUG-P17-2017). All participants gave written informed consent before any evaluation.

### 2.2. Data Collection

#### 2.2.1. Clinical Evaluation

Social, demographic, and clinical data were obtained using a questionnaire applied by trained professionals. Blood pressure was measured using a semiautomatic digital blood pressure monitor (Omron HEM-7200) on the participant’s right arm in a sitting position and after resting for at least 10 min.

#### 2.2.2. Anthropometric and Body Composition Assessment

Body weight and height were measured with the subjects barefoot and wearing light clothing using a digital scale (Seca 769) and a stadiometer (Seca 213-I). WC was measured at the intermediate point between the last rib and the iliac crest using a metal tape (Lufkin W606PM).

Body composition measurements (total fat mass, fat mass percentage, VFA, skeletal muscle mass, and free fat mass) were assessed in subjects with at least 8 h of fasting by multifrequency BIA (InBody S10) (Biospace Co., Ltd., Seoul, Korea). The evaluation was carried out while subjects were sitting by placing adhesive electrodes on hands and feet. On the hands, one electrode was placed over the ulna head, and the other wrapped around the middle finger. On the feet, the upper electrode was on the medial malleolus and the lower electrode at the base of the second toe.

#### 2.2.3. Biochemical Assays

Venous blood samples were collected with at least 8 h of fasting. For fasting glucose (Trinder GOD-POD Spinreact, Girona, Spain), lipid profile (total cholesterol, triglycerides (TGs), and high-density lipoprotein-cholesterol (HDL-c)) (GPO-POD Spinreact, Girona, Spain), enzymatic colorimetric assays were used. Low-density lipoprotein-cholesterol (LDL-c) was calculated using the Friedewald equation [19].

#### 2.2.4. Anthropometric, Adiposity, and Insulin Resistance Indexes

Anthropometric indices were defined and calculated according to the following equations:

BMI = body weight (kg)/height square (m^2^), (1)

WHtR = WC (cm)/height (cm),(2)

AIs were defined and calculated according to established and published [10,11,12,15,16] equations:

VAI (women) = (WC/[36.58 + (1.89 × BMI)]) × (TG (mmol/L)/0.81) × (1.52/HDL-c (mmol/L)),(3)

VAI (men) = WC/[39.68 + (1.88 × BMI)]) × (TG (mmol/L)/1.03) × (1.31/ HDL-c (mmol/L)),(4)

LAP (women) = (WC-58) × TG (mmol/L),(5)

LAP (men) = (WC-65) × TG (mmol/L),(6)

NVAI (women) = 1/[1 + exp [−(−18.765 + (0.058 × Age) + (0.14 × WC) + (0.057 × MBP) + (0.004 × TG (mmol/L)) + (−0.057 × HDL-c (mmol/L)))]](7)

NVAI (men) = 1/[1 + exp [−(−21.858 + (0.099 × Age) + (0.10 × WC) + (0.12 × MBP) + (0.006 × TG (mmol/L)) + (−0.077 × HDL-C (mmol/L)))]],(8)

where the mean blood pressure was calculated as:

MBP = [1/3(systolic blood pressure) + 2/3(diastolic blood pressure)] based on an average of two measurements,(9)

METS-VF = 4.466 + 0.011[(Ln (METS-IR))^3^] + 3.239[(Ln (WHtR))^3^] + 0.319(Sex) + 0.594(Ln (Age)), (10)

where sex was a binary response variable (male = 1, female = 0) and METS-IR was calculated as following:

METS-IR= (Ln ((2 × fasting glucose (mg/dL)) + TG (mg/dL)) × BMI)/(Ln (HDL-c (mg/dL))),(11)

IRIs were defined and calculated according to established and published [13,14] equations:

TyG = Ln [TG (mg/dL) × fasting glucose (mg/dL)/2],(12)

TyG–BMI = TyG × BMI,(13)

TyG–WC = TyG × WC,(14)

#### 2.2.5. Metabolic Syndrome Diagnosis

The Harmonized Joint Scientific Statement on metabolic syndrome was used [20]. Subjects with at least three out of five risk factors were diagnosed with MetS. The risk factors were WC ≥ 94 cm in men and ≥88 cm in women, systolic blood pressure ≥ 130 mmHg and/or diastolic ≥ 85 mmHg, glucose ≥ 100 mg/dL, TG ≥ 150 mg/dL, and HDL-c < 40 mg/dL in men and <50 mg/dL in women.

### 2.3. Statistical Analysis

IBM SPSS Statistics 25.0 software was used for statistical analysis. Results are expressed as the mean ± standard deviation for continuous variables with a normal distribution and as median and interquartile range for variables with a skewed distribution. Categorical variables are expressed as proportions (percentage). Normal distributions were tested by Kolmogorov–Smirnov.

Multiple linear regression was used to develop two sex-specific Mexican adiposity indices (MAIs), using VFA as the dependent variable and anthropometric measurements as independent variables. Binary logistic regression was used to develop two biochemical–anthropometric indices (BAIs) to identify MetS, where MetS was considered the dependent variable, and anthropometric, body composition, and biochemical characteristics as the independent variables.

The indices’ predictive performance and cut-off values to identify CR and MetS were determined using receiver operating characteristic (ROC) curve analysis. Likewise, the area under the curve (AUC) for the proposed indices was compared with those for other indices using ROC curve analysis.

Our proposed indices were validated in the secondary cohort. First, Cohen’s kappa coefficient analysis was performed to assess the concordance between MetS status obtained by BAIs compared to the MetS diagnosis by the harmonized criteria. Secondly, we evaluated the concordance of CR status identified by MAIs compared to CR status identified by the VFA-BIA.

ROC curve analysis was also used to determine the VFA-BIA cut-off value to identify CR in Mexican adults. For this analysis, we examined the number of risk factors (systolic blood pressure ≥ 130 mmHg and/or diastolic ≥ 85 mmHg, glucose ≥ 100 mg/dL, TG ≥ 150 mg/dL, and HDL-c < 40 mg/dL in men and <50 mg/dL in women) in each subject. Sensitivity and specificity for each cut-off value were estimated by detecting the subjects with two or more of these risk factors. Additionally, we analyzed the age and sex-specific VFA cut-off values. Significance was defined as a *p*-value < 0.05 for all analyses.

## 3. Results

### 3.1. Characteristics of the Study Population

Among the 904 participants, 141 (15.6%) were women and 763 (84.4%) were men. The mean age of the subjects was 33.5 ± 8.1 years. A comparative analysis of anthropometric, body composition and clinical and biochemical characteristics between MetS and non-MetS groups is shown in Table 1. The prevalence of MetS was 51.2% (*n* = 463). The MetS group showed a higher prevalence of MetS risk factors and higher values for all the variables including AIs and IRIs. Supplementary Materials Table S1 shows a comparative analysis of the study variables stratified by sex and MetS diagnosis. Additionally, univariate analysis was used to verify whether sex influenced anthropometric body composition and clinical and biochemical variables. There was no effect of sex on these variables.

The general characteristics of the secondary cohort used for indices validation are presented in Supplementary Materials Table S2.

### 3.2. Mexican Adiposity Indices to Identify Cardiometabolic Risk Associated with Visceral Adipose Tissue Accumulation in Mexican Adults

Two sex-specific mathematical models to identify CR associated to VAT accumulation were developed: MAIm for men and MAIw for women. These indices combined height and the interaction between body weight and WC (Table 2). The interaction between these variables was included in MAIs because of multicollinearity in the models. The resulting equations were the following:

MAIm = 333.36 + ([body weight (kg) × WC (cm)] × 0.02) − (height (cm) × 2.10),(15)

MAIw = 249.19 + ([body weight (kg) × WC (cm)] × 0.02) − (height (cm) × 1.60).(16)

The *R*^2^ values were 0.83 and 0.86, respectively, and both models were statistically significant.

**Figure 2:**
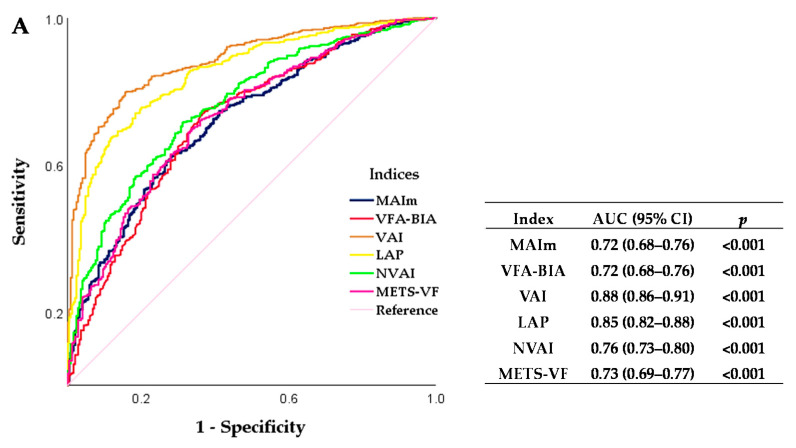

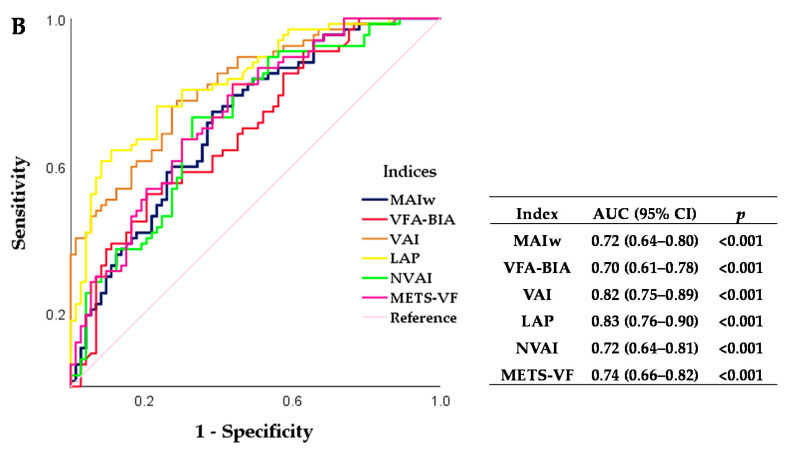
ROC curve and AUC comparison of MAIs and AIs to identify CR associated to VAT accumulation in Mexican adults. (**A**) MAIm and (**B**) MAIw ROC curves to identify CR associated with VAT accumulation. A *p*-value ≤ 0.05 was taken as statistically significant. ROC: receiver operating characteristic, AUC: area under the curve, CI: confidence interval, MAIm: Mexican adiposity index for men, MAIw: Mexican adiposity index for women, VFA-BIA: visceral fat area as assessed by bioelectrical impedance analysis, VAI: visceral adiposity index, LAP: lipid accumulation product, NVAI: new visceral adiposity index, and METS-VF: metabolic score for visceral fat.

In addition, to determine MAIs’ predictive performance to identify CR associated to VAT accumulation, we compared their AUC with other Ais’ AUC. MAIm presented an AUC = 0.72 (95% CI = 0.68–0.76) and MAIw an AUC = 0.72 (95% CI = 0.64–0.80). The cut-off values to identify CR were for MAIm ≥ 97.7 (AUC = 0.72, sensitivity = 65.6%, and specificity = 65.6%) and for MAIw ≥ 106.3 (AUC = 0.72, sensitivity = 65.7%, and specificity = 64.4%) (Figure 2).

A comparative analysis showed that MAIm in the MetS group presented higher values than the non-MetS group (119.3 ± 29.1 vs. 87.5 ± 23.6, *p* < 0.001, respectively). The same result was observed for MAIw, and the MetS group presented higher values than the non-MetS group (134.9 ± 31.9 vs. 98.9 ± 29.8, *p* < 0.001, respectively).

Validation analysis of MAIs was carried out in the secondary cohort. We found Cohen’s kappa coefficients of 0.62 and 0.73 for MAIm and MAIw, respectively. In the agreement analysis to identify CR associated to VAT accumulation by MAIs and BIA, the correct classification percentage for MAIm was 81.6% and 87.3% for MAIw.

The MAIs comparative analysis in the secondary cohort is presented in Supplementary Materials Table S3 and Figure S1.

### 3.3. The Cut-Off Values for the Visceral Fat Area as Assessed by Bioelectrical Impedance Analysis to Identify Cardiometabolic Risk in Mexican Adults

Several VFA-BIA cut-off values to identify CR in Mexican adults were analyzed by ROC curve analysis (Table 3). The selected cut-off value of 100.3 cm^2^ had the highest sensitivity and specificity values to identify CR in Mexican adults with 66.8% and 64.4%, respectively. The AUC value of the ROC curve analysis was 0.71, CI 95% (0.67–0.74). Based on this cut-off value, a larger incidence of VFA ≥ 100.3 cm^2^ was observed in the MetS group 77.1% (*n* = 357) than the non-MetS group 33.6% (*n* = 148), *p* < 0.001.

Furthermore, age and sex-specific cut-off values of VFA-BIA were determined. Based on the age classification used by Matsushita et al. [21], we classified subjects into two groups (20–40 and 41–60-years). VFA cut-off values were smaller in the 20–40 year group for both sexes (97.0 cm^2^ for men and 103.7 cm^2^ for women) compared with the 41–60 year group (103.6 cm^2^ for men and 115.6 cm^2^ for women). However, for women classified in the 41–60 year group, the AUC value of the ROC curve analysis was not statistically significant (Supplementary Materials Table S4).

### 3.4. Biochemical–Anthropometric Indices to Identify Metabolic Syndrome in Mexican Adults

Two mathematical models to identify MetS were developed with the following variables: TyG, BMI, and MBP (BAI1) and TyG, VFA, and MBP (BAI2) (Table 4). These models were chosen because they had the highest Nagelkerke coefficient *R*^2^ of 0.64 and 0.63 for BAI1 and BAI2, respectively. In addition, the BAI1 and BAI2 classified 84.5% and 84.2% of the subjects with MetS diagnosis, respectively. The resulting equations were:

BAI1 = 1/[1 + e − [−43.38 + (3.18 × TyG) + (0.29 × BMI (kg/m^2^)) + (0.08 × MBP (mmHg))]], (17)

BAI2 = 1/[1 + e − [−39.11 + (3.20 × TyG) + (0.03 × VFA (cm^2^)) + (0.08 × MBP (mmHg))]], (18)

**Figure 3:**
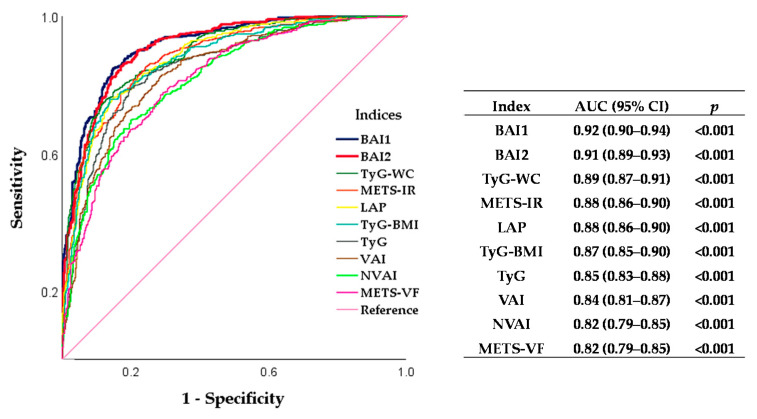
ROC curve analysis and AUC comparison of BAIs, AIs, and IRIs to identify MetS in Mexican adults. The AUC values of BAIs, AIs, and IRIs are presented in descending order. A *p*-value ≤ 0.05 was taken as statistically significant. ROC: receiver operating characteristic, AUC: area under the curve, BAI: biochemical–anthropometric index, TyG–WC: Triglycerides–glucose waist circumference index, METS-IR: metabolic score for insulin resistance, LAP: lipid accumulation product, TyG–BMI: triglycerides–glucose body mass index, TyG: triglycerides–glucose index, VAI: visceral adiposity index, NVAI: new visceral adiposity index, and METS-VF: metabolic score for visceral fat.

To determine the predictive performance of BAIs to identify MetS, we compared their AUC with other IRIs’ AUCs (Figure 3). We observed the highest performance for BAI1 (AUC = 0.92, 95% CI = 0.90–0.93) and BAI2 (AUC = 0.91, 95% CI = 0.89–0.93). The cut-off value for BAI1 was 0.5 (sensitivity = 84.7% and specificity = 84.8%), and for BAI2 it was 0.5 (sensitivity = 83.8% and specificity = 83.9%).

A comparative analysis showed that BAI1 in the MetS group presented higher values than the non-MetS group (0.9 (0.3) vs. 0.1 (0.3), *p* < 0.001, respectively). The same result was observed for BAI2. The MetS group presented higher values than the non-MetS group (0.9 (0.3) vs. 0.1 (0.3), *p* < 0.001, respectively).

Validation analysis of BAIs was carried out in the secondary cohort. We found a Cohen’s kappa coefficient of 0.69 and 0.67 for BAI1 and BAI2, respectively. In the agreement analysis to identify MetS by BAIs and harmonized criteria, the correct classification percentage for BAI1 was 84.9% and for BAI2 was 83.9%. Supplementary Materials Figure S2 shows the ROC curve analysis and AUC comparison of BAI1 and BAI2 to identify MetS in the secondary cohort.

The BAIs’ comparative analysis in the secondary cohort is presented in Supplementary Materials Table S2.

Finally, we also compared the predictive performance of MAIs, BAIs, and common anthropometric indicators (BMI, WC, and WHtR) to identify CR in Mexican adults. According to the sensitivity and specificity, MAIs and BAIs were better predictors than BMI, WC, and WHtR. BAIs showed higher AUC (0.89), sensitivity (BAI1 = 80.6% and BAI2 = 82.0%), and specificity (BAI1 = 80.1% and BAI2 = 81.6%) (Supplementary Materials Table S5).

## 4. Discussion

In this cross-sectional study in Mexican adults, two sex-specific MAIs to identify CR and two BAIs to identify MetS were proposed. The MAIs were developed including only anthropometric variables. Since sex has an important influence on the variability of VAT, an equation for each sex was included. Both MAIs were validated and showed to be good predictors of CR associated to VAT accumulation. The BAIs were developed including variables related to IR and VAT because these risk factors have been related to MetS incidence. Both BAIs were the best predictors of MetS in comparison with other AIs and IRIs. Additionally, a cut-off value of VFA-BIA to identify CR in Mexican adults was proposed, and it was similar to the cut-off value proposed by JSSO.

According to the National Health and Nutrition Survey, the prevalence of MetS in Mexican adults increased 15.3% from 2006 to 2016 [18]. Recently, a systematic meta-analysis of MetS prevalence in apparently healthy Mexican adults showed an estimated MetS prevalence of 41% [22]. In addition, the proportion of subjects with at least one MetS component was 95.3%. Among these components, one of the most frequently found was the presence of abdominal obesity [18]. These epidemiological data indicate that the current health status in Mexicans is alarming. Therefore, this study looked for additional strategies to support the necessity of attending to the increasing MetS incidence and the CR associated with VAT accumulation in the Mexican population.

### 4.1. Sex-Specific Mexican Adiposity Indices and Visceral Fat Area Cut-Off Value Assessed by Bioelectrical Impedance Analysis to Identify Cardiometabolic Risk Associated with Visceral Adipose Tissue Accumulation in Mexican Adults

VAT accumulation has a strong association with CR. However, VAT distribution differs between men and women due to the influence of sex hormones on adipose tissue function and metabolism. Thus, associations of VAT with CR may differ between men and women [23]. For this reason, it was important to develop sex-specific MAIs. Although most participants were men, MAIs are helpful in both sexes, since MAIm and MAIw proved to be good predictors of CR. However, we highlighted the importance of validating MAIw in a larger cohort.

Our equations only include anthropometric variables, making them accessible, easy, and quick to use in daily clinical practice, an advantage over other AIs. To our knowledge, the MAIs are the first indices developed for Mexicans to identify CR associated with VAT accumulation. Although METS-VF was previously developed for the Mexican population, it was created to identify T2DM and hypertension [16].

Other AIs (VAI, LAP, NVAI, and METS-VF) showed the best predictive performance to identify CR (defined as having two risk factors). However, MAIs presented the most approximated AUC value to VFA-BIA, meaning that MAIs consider the influence of VAT accumulation on CR.

Additionally, a cut-off value of 100.3 cm^2^ for VFA-BIA was proposed to identify Mexican adults who could have CR at some point. Several cut-off values for VFA have mainly been established in Asian populations [24]. When comparing the cut-off value obtained in this work (100.3 cm^2^, sensitivity = 66.8% and specificity = 64.4%) with the reference cut-off value established by the JSSO, we found similar values (100 cm^2^, sensitivity = 69% and specificity = 62%) [6]. Therefore, this cut-off value for Mexican adults is validated.

On the other hand, VFA cut-off values stratified by sex and age to identify CR were proposed. Consistent with Matsushita et al. [21], our VFA cut-off values showed that as age increased, the VFA cut-off value increased as well. Nonetheless, the number of subjects in all groups (except men 20–40 years old) was small. Therefore, these VFA cut-off values need to be validated in a larger Mexican cohort.

Based on the cut-off values of MAIs and VFA-BIA, it was observed that women with more visceral fat were less likely to develop CR than men. In women, fat is stored in a more significant proportion in the subcutaneous adipose tissue and, consequently, the lipolytic rate is higher in this tissue, while in men, lipolysis is mainly conducted in VAT, since this is the main store of fat in this sex [25]. Furthermore, estrogens play an important role in stimulating preadipocyte proliferation in subcutaneous adipose tissue [26]. Taking together these mechanisms, premenopausal women have less CR than men [27].

### 4.2. Biochemical–Anthropometric Indices to Identify Metabolic Syndrome

In this study, more than half (51.2%) of the subjects presented MetS, and the most prevalent components were reduced HDL-c (90.3%), elevated WC (81.6%), and TG (77.5%). The same trend was observed in the non-MetS group, consistent with Campos-Nonato et al. [18]. Likewise, subjects with a VFA ≥ 100.3 cm^2^ were high in both groups (MetS = 77.1% and non-MetS = 33.6%). Hence, subjects without MetS diagnosis could already have CR according to the VFA cut-off value proposed in this study. For this reason, two affordable and easy-to-use BAIs to support the early diagnosis of MetS were proposed.

Since VAT and IR play a central role in the physiopathology of MetS [28], BAI1 includes TyG index, BMI, and MBP, and BAI2 includes TyG, VFA, and MBP. VFA was used as an indicator of VAT accumulation, and the TyG index was used as a marker of IR, which may help diagnose MetS.

When BAI1 and BAI2 were compared with several AIs and IRIs, they showed to be better predictors of MetS in our study population. These results agree with the importance of developing specific indices tailored to the study population. The high performance of BAIs for MetS identification suggests that they could be proposed as clinical tools.

Several studies have established differences between AIs and IRIs for the MetS diagnosis and CR. NVAI and METS-VF were developed in specific populations (Korean and Mexican, respectively) as alternatives to the previously reported IAs and IRIs, since they were less accurate in identifying CR in their respective populations [15,16]. Therefore, ethnicity should be considered to develop specific indices to identify MetS, knowing that body composition and metabolic profile differ between ethnicities [29].

Therefore, population-specific cut-off values for AIs and IRIs to identify MetS have been proposed. For instance, in a study with a Turkish population with a BMI > 25 kg/m^2^ and 18–65 years old, a VAI cut-off value of 2.21 has been proposed [30]. A similar value of 2.23 was found in the general Italian population between 30 and 41 years old [31]. In the Venezuelan population of 30–49 years composed of different ethnicities, Hispanic white, afro-Venezuelan, and Amerindian, different cut-off values were shown at 1.88, 1.35, and 1.86, respectively [32].

In middle-aged Korean and Chinese populations, LAP was a good predictor of MetS compared to VAI, TyG, and WHtR [33,34]. In healthy Argentinian subjects between 18 and 65 years, LAP was also a good predictor of MetS [35].

In the same study, Okosun et al. found that LAP for Mexican Americans and TyG–WC for non-Hispanic white and non-Hispanic black were the best predictors of MetS [36].

### 4.3. Advantages and Limitations

This study is the first to propose a cut-off value to identify CR associated to VAT accumulation in Mexican adults. The closeness between our VFA cut-off value of 100.3 cm^2^ and 100 cm^2^ obtained by JSSO suggests consistency of this measurement between different populations. However, more studies are needed to validate the VFA cut-off value in other populations.

Our indices seem to be affordable and useful tools for daily clinical practice in primary health. In Mexico, imaging methods are mainly available at tertiary healthcare facilities, and they are not usually employed to assess VFA. For this reason, we developed population-specific indices (MAIs and BAIs) that could help identify Mexicans subjects at risk of cardiometabolic diseases.

Since our indices were modeled with data from Mexican adults, these findings cannot be applied to other populations. In addition, we did not analyze factors related to lifestyle and its impact on CR and MetS development.

The proposed indices in our study cannot predict T2DM or other chronic diseases. The main purpose of this study was to identify subjects at risk of cardiometabolic diseases, then subjects with known chronic conditions were not included. However, according to Mexican guidelines, some subjects were identified with hypertension after a clinical evaluation (17.3%) [37]. Hence, MAIs’ and BAIs’ predictive performance for hypertension was evaluated, and it was found that they were good predictors (Supplementary Materials Figure S3). In addition, the VFA cut-off value proposed in this study was analyzed to identify hypertension. Even though it showed a moderate sensitivity (77.6%), the specificity was low (49.1%). Thus, further studies in larger cohorts or prospective designs are necessary to evaluate whether our indices could predict T2DM and hypertension.

Another limitation was the cross-sectional design, and then we cannot demonstrate the causality of the evaluated risk factors on MetS development. The number of women included in this study was relatively small in comparison to the total sample. Moreover, when stratified by sex and age to determine VFA cut-off values to identify CR, some groups had a small sample size. Hence, further studies are needed in a large representative sample of the general Mexican population.

Finally, VFA measurement was not compared to imaging methods known as the gold standard (magnetic resonance imaging or computed tomography scan); however, several authors have found that VFA measured by BIA has a good correlation with imaging methods [38,39,40,41,42].

## 5. Conclusions

We proposed and validated MAIs and BAIs as additional clinical tools to identify CR associated to VAT accumulation and MetS in Mexican adults, respectively. Thus, the importance of developing population-specific indices was highlighted. Additionally, we found a VFA cut-off value of 100.3 cm^2^, which was similar to the report by the JSSO. Thus, it could be a reliable indicator to identify CR in Mexican adults.

## Figures and Tables

**Table 1:** Comparison of anthropometric, body composition, and clinical and biochemical characteristics stratified by MetS diagnosis.

Variable	Non-MetS *n* = 441	MetS *n* = 463	*p*
Age (years)	32.0 ± 7.6	34.9 ± 8.2	<0.001
Male (%)	353 (80.0)	410 (88.6)	<0.001
BMI (kg/m^2^)	25.8 ± 3.4	29.9 ± 3.7	<0.001
WHtR	0.5 ± 0.1	0.6 ± 0.1	<0.001
WC (cm)	89.1 ± 8.8	100.1 ± 9.0	<0.001
VFA (cm^2^)	89.2 ± 31.4	121.8 ± 30.5	<0.001
Total body fat (kg)	18.6 ± 7.0	26.5 ± 7.6	<0.001
Body fat percentage (%)	25.1 ± 7.4	30.6 ± 6.5	<0.001
Free fat mass (kg)	54.6 ± 8.3	59.2 ± 8.4	<0.001
Skeletal muscle mass (kg)	30.9 ± 5.1	33.6 ± 5.0	<0.001
Systolic BP (mmHg)	118.3 ± 11.7	128.5 ± 14.0	<0.001
Diastolic BP (mmHg)	73.7 ± 8.8	81.5 ± 10.2	<0.001
MBP (mmHg)	88.6 ± 8.5	97.2 ± 10.3	<0.001
FBG (mg/dL)	89.9 ± 10.5	99.6 ± 12.5	<0.001
Total cholesterol (mg/dL)	175.9 ± 33.9	191.0 ± 33.3	<0.001
HDL-c (mg/dL)	40.3 ± 9.1	33.7 ± 6.9	<0.001
LDL-c (mg/dL)	110.1 ± 28.5	113.9 ± 31.3	0.058
VLDL-c (mg/dL)	25.5 ± 13.1	43.7 ± 18.9	<0.001
TG (mg/dL)	127.3 ± 65.2	218.6 ± 94.5	<0.001
VAI	1.8 (1.3)	3.7 (2.5)	<0.001
LAP	37.2 ± 23.2	87.9 ± 43.2	<0.001
NVAI	0.7 (0.5)	1.0 (0.1)	<0.001
METS-VF	6.4 ± 0.6	7.0 ± 0.4	<0.001
TyG	8.5 ± 0.5	9.2 ± 0.5	<0.001
TyG–BMI	220.7 ± 32.7	274.5 ± 35.1	<0.001
TyG–WC	760.1 ± 97.6	921.2 ± 94.6	<0.001
METS-IR	40.4 ± 6.5	51.5 ± 7.3	<0.001
MetS components, *N* (%)			
Elevated WC	128 (29.0)	378 (81.6)	<0.001
Elevated BP	73 (16.6)	284 (61.3)	<0.001
Reduced HDL-c level	237 (53.7)	418 (90.3)	<0.001
Elevated FBG level	54 (12.2)	256 (55.3)	<0.001
Elevated TG level	103 (23.4)	359 (77.5)	<0.001

Data are presented as the mean ± standard deviation or median and interquartile range. Comparisons were determined by *t*-student for independent samples or U Mann–Whitney. Categorical variables are presented as the count (percentage), and comparisons were determined by χ² test or Fischer’s exact test. A *p*-value ≤ 0.05 was taken as statistically significant. MetS: metabolic syndrome, BMI: body mass index, WHtR: waist-to-height ratio, WC: waist circumference, VFA: visceral fat area, BP: blood pressure, MBP: mean blood pressure, FBG: fasting blood glucose, HDL-c: high-density lipoprotein cholesterol, LDL-c: low-density lipoprotein cholesterol, VLDL-c: very-low-density lipoprotein cholesterol, TGs: triglycerides, VAI: visceral adiposity index, LAP: lipid accumulation product, NVAI: New visceral adiposity index, METS-VF: Metabolic score for visceral fat, TyG: triglycerides–glucose index, TyG–BMI: triglycerides–glucose body mass index, TyG–WC: triglycerides–glucose waist circumference index, and METS-IR: metabolic score for insulin resistance.

**Table 2:** Multiple linear regression analysis models (MAIs) to identify CR associated to VAT accumulation in Mexican adults.

Variable	Non-Standardized Coefficient B	Standardized Coefficient β	Significance	*t*	*R* ^2^	F	*p*
MAIm					0.83	1877.23	<0.001
Body weight * WC (kg * cm)	0.02	0.95	<0.001	61.02			
Height (cm)	−2.10	−0.36	<0.001	−23.10			
MAIw					0.86	409.46	<0.001
Body weight * WC (kg * cm)	0.02	0.92	<0.001	28.32			
Height (cm)	−1.60	−0.21	<0.001	−6.52			

Multiple linear regression models were used to associate VFA as the dependent variable with anthropometric measurements as the independent variables. A *p*-value ≤ 0.05 was taken as statistically significant. MAIm: Mexican adiposity index for men, MAIw: Mexican adiposity index for women, and WC: waist circumference.

**Table 3:** The cut-off values of VFA-BIA to identify CR in Mexican adults.

	Cut-Off	Sensitivity (%)	Specificity (%)	AUC	95% CI	*p*
				0.71	(0.67–0.74)	<0.001
VFA (cm^2^)	95.1	73.7	59.2			
	100.3	66.8	64.4			
	105.1	58.7	69.0			

The optimal cut-off value was obtained as the maximum sensitivity and specificity. A *p*-value ≤ 0.05 was taken as statistically significant. VFA: visceral fat area and AUC: area under the curve. 95% CI = 95% confidence interval.

**Table 4:** Logistic regression models (BAIs) to identify MetS in Mexican adults.

Variable	Standardized Coefficient β	Wald Statistic	*p*	OR	95% CI	*p*
BAI1						<0.001
TyG	3.18	157.01	<0.001	23.99	(14.59–39.43)	
BMI (kg/m^2^)	0.29	83.47	<0.001	1.34	(1.25–1.42)	
MBP (mmHg)	0.08	46.57	<0.001	1.08	(1.06–1.10)	
BAI2						<0.001
TyG	3.20	162.20	<0.001	24.46	(14.96–40.01)	
VFA (cm^2^)	0.03	79.21	<0.001	1.03	(1.02–1.04)	
MBP (mmHg)	0.08	51.12	<0.001	1.08	(1.06–1.11)	

Binary logistic regression models were used to associate MetS diagnosis as the dependent variable with anthropometric, body compositions, and clinical estimators as the independent variables. A *p*-value ≤ 0.05 was taken as statistically significant. BAI: biochemical–anthropometric index, TyG: triglycerides–glucose index, BMI: body mass index, MBP: mean blood pressure, VFA: visceral fat area, and OR: odds ratio. 95% CI = 95% confidence interval.

## Data Availability

All data presented in this study are available on request from the corresponding author.

## Supplementary Materials

The following is available online at https://www.mdpi.com/article/10.3390/healthcare9111561/s1, Table S1: Comparison of anthropometric, body composition, and clinical and biochemical characteristics stratified by sex and MetS diagnosis; Table S2: Comparison of anthropometric, body composition and biochemical characteristics in the secondary cohort stratified by MetS diagnosis; Table S3: Comparison of MAIs in secondary cohort stratified by sex and MetS diagnosis; Table S4: Sex and Age-specific cut-off values of VFA-BIA to identify CR in Mexican adults; Table S5: Predictive performance of MAIs, BAIs, and indicators of interest to identify CR in Mexican adults; Figure S1: ROC curve and AUC of MAIm and MAIw to identify CR associated to VAT accumulation in the secondary cohort; Figure S2: ROC curve and AUC comparison of BAI1 and BAI2 to identify MetS in the secondary cohort; Figure S3: ROC curve analysis and AUC comparison of BAIs, MAIs, and METS-VF to identify hypertension in Mexican adults.
