# Supplementary material for: Novel Adiposity and Biochemical–Anthropometric Indices to Identify Cardiometabolic Risk and Metabolic Syndrome in Mexican Adults
Source: Healthcare (Basel). 2021 Nov 16;9(11):1561. doi: 10.3390/healthcare9111561 (PMC8620800; doi:10.3390/healthcare9111561)
Supplement: Supplementary file 1 [file healthcare-09-01561-s001.zip › healthcare-1433494-supplementary.pdf]

healthcare

MDPI

Table S1. Comparison of anthropometric, body composition, clinical and biochemical characteristics stratified by sex and MetS diagnosis.

Variable	Men n = 763			Women n = 141		
	Non-MetS n = 353	MetS n = 410	*p*	Non-MetS n = 88	MetS n = 53	*p*
Age (years)	32.0 ± 7.8	34.7 ± 8.3	< 0.001	31.8 ± 7.0	36.2 ± 7.4	0.001
BMI (kg/m²)	25.9 ± 3.2	29.8 ± 3.5	< 0.001	25.4 ± 4.0	30.8 ± 4.6	< 0.001
WHtR	0.5 ± 0.1	0.6 ± 0.1	< 0.001	0.5 ± 0.1	0.6 ± 0.1	< 0.001
WC (cm)	89.8 ± 8.1	100.5 ± 8.9	< 0.001	87.0 ± 10.7	97.0 ± 8.8	< 0.001
VFA (cm²)	86.9 ± 30.3	120.1 ± 29.7	< 0.001	98.4 ± 34.2	135.1 ± 33.0	< 0.001
Total body fat (kg)	17.9 ± 6.6	25.9 ± 7.4	< 0.001	21.5 ± 8.1	30.5 ± 8.4	< 0.001
Body fat percentage (%)	23.4 ± 6.4	29.5 ± 5.7	< 0.001	32.2 ± 7.1	39.0 ± 6.1	< 0.001
Free fat mass (kg)	57.4 ± 6.5	60.8 ± 7.2	< 0.001	43.4 ± 4.4	46.5 ± 5.1	< 0.001
Skeletal muscle mass (kg)	32.6 ± 4.0	34.6 ± 4.3	< 0.001	23.9 ± 2.7	25.9 ± 3.1	< 0.001
Systolic BP (mmHg)	119.5 ± 11.9	129.3 ± 13.8	< 0.001	113.6 ± 10.0	122.0 ± 14.0	< 0.001
Diastolic BP (mmHg)	73.9 ± 8.9	81.6 ± 10.1	< 0.001	73.1 ± 8.7	80.4 ± 10.9	< 0.001
MBP (mmHg)	89.1 ± 8.6	97.5 ± 10.1	< 0.001	86.6 ± 8.1	94.2 ± 10.8	< 0.001
FBG (mg/dL)	90.0 ± 10.6	99.8 ± 12.4	< 0.001	89.4 ± 10.2	98.3 ± 13.1	< 0.001
Total cholesterol (mg/dL)	177.8 ± 35.4	190.9 ± 33.4	< 0.001	168.1 ± 26.3	192.0 ± 32.7	< 0.001
HDL-C (mg/dL)	39.4 ± 8.5	33.1 ± 6.6	< 0.001	43.9 ± 10.5	38.6 ± 6.6	< 0.001
LDL-C (mg/dL)	111.4 ± 29.6	113.3 ± 31.4	< 0.001	105.1 ± 22.8	118.3 ± 30.1	0.008
VLDL-C (mg/dL)	27.0 ± 13.7	44.8 ± 18.7	< 0.001	17.0 (10.3)	30.0 (17.8)	< 0.001
TG (mg/dL)	135.0 ± 68.4	224.1 ± 93.3	< 0.001	86.5 (53.0)	150.0 (91.0)	< 0.001
VAI	1.8 (1.2)	3.8 (2.4)	< 0.001	2.0 ± 1.1	4.0 ± 2.3	< 0.001
LAP	38.9 ± 23.8	89.2 ± 43.3	< 0.001	28.1 (24.3)	68.8 (40.0)	< 0.001
NVAI	0.7 (0.5)	1.0 (0.1)	< 0.001	0.5 ± 0.3	0.8 ± 0.2	< 0.001
METS-VF	6.5 ± 0.5	7.0 ± 0.4	< 0.001	6.2 ± 0.6	6.8 ± 0.3	< 0.001
TyG	8.6 ± 0.5	9.2 ± 0.4	< 0.001	8.3 ± 0.4	8.9 ± 0.5	< 0.001
TyG-BMI	223.2 ± 31.3	274.4 ± 34.1	< 0.001	210.5 ± 36.2	274.9 ± 42.5	< 0.001
TyG-WC	773.3 ± 87.3	927.0 ± 90.5	< 0.001	713.8 ± 102.4	866.6 ± 86.9	< 0.001
METS-IR	40.9 ± 6.2	51.7 ± 7.2	< 0.001	38.1 ± 6.9	49.8 ± 7.8	< 0.001
MetS components, N (%)						
Elevated WC	95 (26.9)	332 (81.0)	< 0.001	33 (37.5)	46 (86.8)	< 0.001
Elevated BP	65 (18.4)	256 (62.4)	< 0.001	8 (9.1)	28 (52.8)	< 0.001
Reduced HDL-C level	172 (48.7)	367 (89.5)	< 0.001	65 (73.9)	51 (96.2)	0.001
Elevated FBG level	44 (12.5)	230 (56.1)	< 0.001	10 (11.4)	26 (49.1)	< 0.001
Elevated TG level	97 (27.5)	332 (81.0)	< 0.001	6 (6.8)	27 (50.9)	< 0.001
VFA ≥ 100 cm², N (%)						
	107 (30.3)	315 (76.8)	< 0.001	41 (46.6)	42 (79.2)	< 0.001

Data are presented as mean ± standard deviation or median and interquartile range. Comparisons were determined by t-student for independent samples or U-Mann-Whitney. Categorical variables are presented as count (percentage) and comparisons were determined by χ^2 test or Fischer's exact test. A *p*-value ≤ 0.05 was taken as statistically significant. Abbreviations: MetS: Metabolic syndrome, BMI: Body mass index, WHtR: Waist to height ratio, WC: Waist circumference, VFA: Visceral fat area, BP: Blood pressure, MBP: Mean blood pressure, FBG: Fasting blood glucose, HDL-C: High-density lipoprotein cholesterol, LDL-C: Low-density lipoprotein cholesterol, VLDL-C: Very-low-density lipoprotein cholesterol, TG: Triglycerides, VAI: Visceral adiposity index, LAP: Lipid accumulation product, NVAI: New visceral adiposity index, METS-VF: Metabolic score for visceral fat, TyG: Triglycerides-glucose index, TyG-BMI: Triglycerides-glucose Body mass index, TyG-WC: Triglycerides-glucose Waist circumference index and METS-IR: Metabolic score for insulin resistance.

Table S2. Comparison of anthropometric, body composition and biochemical characteristics in secondary cohort stratified by MetS diagnosis.

Variable	Non-MetS n = 73	MetS n = 113	*p*
Age (years)	38.0 ± 10.7	40.3 ± 10.4	0.160
Male (%)	34 (46.6)	72 (63.7)	0.021
BMI (kg/m^2)	25.7 ± 4.2	30.6 ± 4.4	< 0.001
WC (cm)	87.6 ± 11.4	101.8 ± 11.2	< 0.001
Visceral fat area (cm^2)	89.7 ± 45.6	137.5 ± 50.9	< 0.001
Total body fat (kg)	20.8 ± 8.3	30.4 ± 9.1	< 0.001
Body fat percentage (%)	30.2 ± 8.6	35.7 ± 7.4	< 0.001
Free fat mass (kg)	47.3 ± 9.1	54.3 ± 10.2	< 0.001
Skeletal muscle mass (kg)	26.4 ± 5.6	30.6 ± 6.1	< 0.001
Systolic BP (mmHg)	115.8 ± 11.3	126.3 ± 15.2	< 0.001
Diastolic BP (mmHg)	71.3 ± 9.1	78.8 ± 9.0	< 0.001
MBP (mmHg)	86.1 ± 8.7	94.6 ± 10.3	< 0.001
FBG (mg/dL)	94.0 (13.0)	108.0 (12.0)	< 0.001
HDL-C (mg/dL)	49.6 ± 12.0	37.5 ± 8.3	< 0.001
TG (mg/dL)	101.0 (61.0)	173.0 (115.0)	< 0.001
BAI1	0.1 (0.3)	0.9 (0.3)	< 0.001
BAI2	0.1 (0.2)	0.9 (0.4)	< 0.001

Data are presented as mean ± standard deviation or median and interquartile range. Comparisons were determined by t-student for independent samples or U-Mann-Whitney. Categorical variables are presented as count (percentage) and comparisons were determined by χ^2 test or Fischer's exact test. A *p*-value ≤ 0.05 was taken as statistically significant. Abbreviations: MetS: Metabolic syndrome, BMI: Body mass index, WC: Waist circumference, BP: Blood pressure, MBP: Mean blood pressure, FBG: Fasting blood glucose, HDL-C: High-density lipoprotein cholesterol and BAI: Biochemical-anthropometrical index.

Table S3. Comparison of MAIs in secondary cohort stratified by sex and MetS diagnosis.

Variable	Men n = 103			Women n = 79		
	Non-MetS n = 32	MetS n = 71	*p*	Non-MetS n = 38	MetS n= 41	*p*
MAIm	86.4 ± 24.3	127.8 ± 35.0	< 0.001	-	-	-
MAIw	-	-	-	102.7 ± 39.3	137.3 ± 38.7	< 0.001

Data are presented as mean ± standard deviation. Comparisons were determined by t-student for independent samples. A *p*-value ≤ 0.05 was taken as statistically significant. Abbreviations: MetS: Metabolic syndrome, MAIm: Mexican adiposity index for men and MAIw: Mexican adiposity index for women.

Table S4. Sex and Age-specific cut-off values of VFA-BIA to identify CR in Mexican adults.

Age (years)	VFA				*p*
	Cut-off Value (cm²)	Sensitivity (%)	Specificity (%)	AUC	
Men (n = 763)					
20-40 (n = 618)	97.0	67.1	67.3	0.70	< 0.001
41-60 (n = 145)	103.6	76.4	69.2	0.79	< 0.001
Women (n = 141)					
20-40 (n = 112)	103.7	62.0	61.3	0.71	< 0.001
41-60 (n = 29)	115.6	61.1	54.5	0.58	0.500

A *p*-value ≤ 0.05 was taken as statistically significant. Abbreviations: VFA: visceral fat area and AUC: area under the curve.

Table S5. Predictive performance of MAIs, BAIs and indicators of interest to identify CR in Mexican adults.

Indicator	AUC	Sensitivity (%)	Specificity (%)	*p*
**MAIw**	0.72	65.7	64.4	< 0.001
**MAIm**	0.72	65.6	65.6	< 0.001
**BAI1**	0.89	80.6	80.1	< 0.001
**BAI2**	0.89	82.0	81.6	< 0.001
**METS-VF**	0.74	68.5	68.1	< 0.001
**WHtR**	0.71	64.2	63.8	< 0.001
**WC**	0.71	63.7	64.1	< 0.001
**BMI**	0.71	63.8	63.8	< 0.001

A *p*-value ≤ 0.05 was taken as statistically significant. Abbreviations: AUC: area under the curve, MAIw: Mexican adiposity index for women, MAIm: Mexican adiposity index for men, BAI: Biochemical-anthropometric index, METS-VF: Metabolic score for visceral fat, WHtR: Waist to height ratio, WC: Waist circumference and BMI: Body mass index.

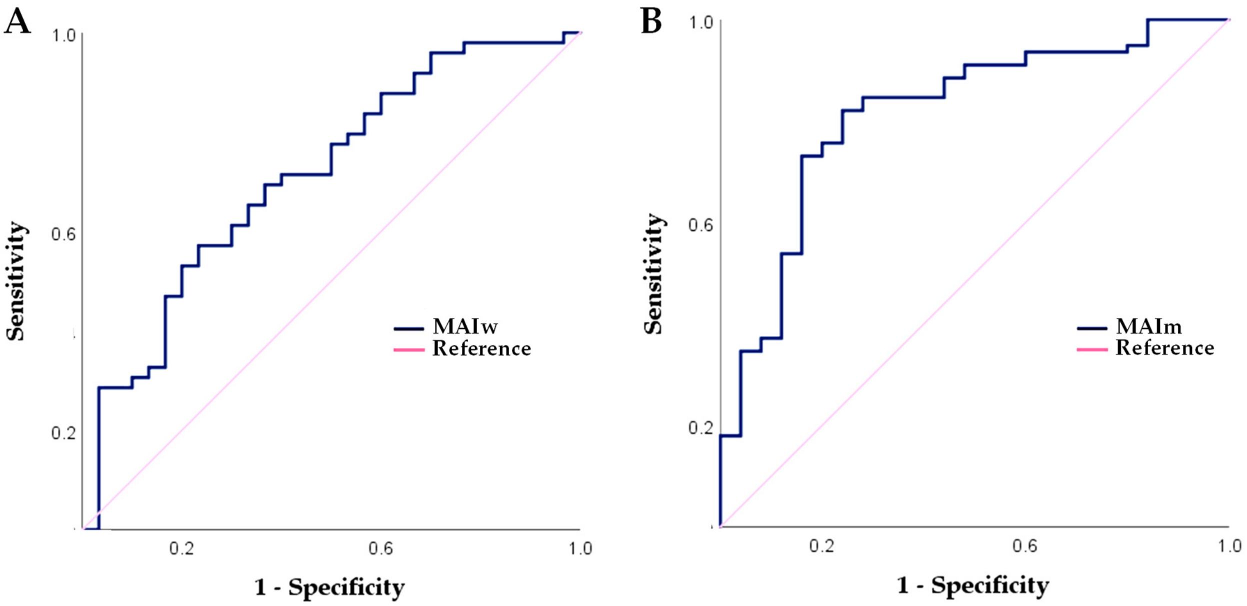

Index	AUC (95% CI)	*p*
MAIw	**0.71 (0.59-0.83)**	**0.002**

Index	AUC (95% CI)	*p*
MAIm	**0.82 (0.72-0.91)**	**<0.001**

Figure S1. ROC curve and AUC of MAIm and MAIw to identify CR associated to VAT accumulation in secondary cohort. (**A**) MAIm and (**B**) MAIw ROC curve to identify CR associated with VAT accumulation. A *p*-value ≤ 0.05 was taken as

statistically significant. Abbreviations: ROC: Receiver operating characteristic, AUC: Area under the curve, CI: Confidence interval, MAIm: Mexican adiposity index for men and MAIw: Mexican adiposity index for women.

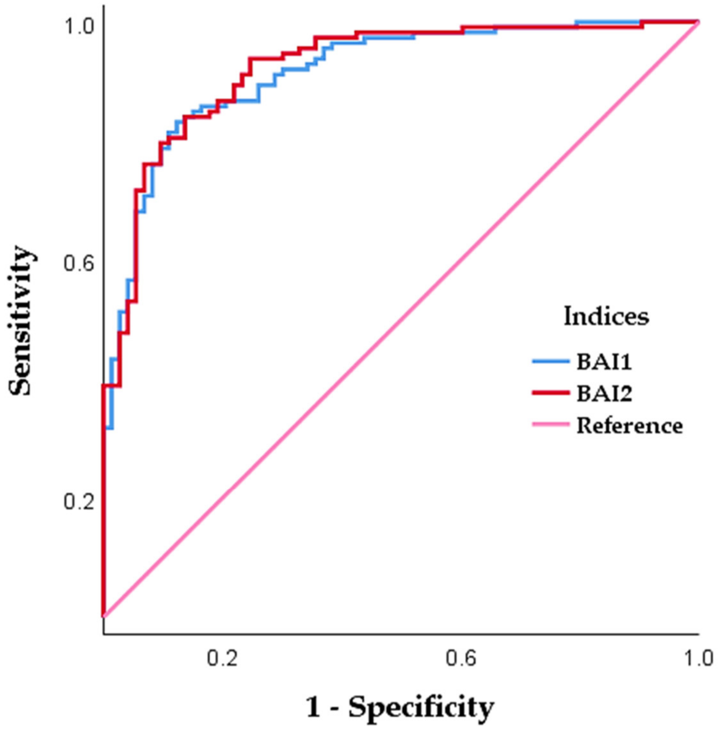

Index	AUC (95% CI)	*p*
BAI1	0.92 (0.88-0.96)	<0.001
BAI2	0.92 (0.88-0.96)	<0.001

Figure S2. ROC curve and AUC comparison of BAI1 and BAI2 to identify MetS in secondary cohort. A *p*-value ≤ 0.05 was taken as statistically significant. Abbreviations: ROC: Receiver operating characteristic, AUC: Area under the curve, CI: Confidence interval and BAI: Biochemical-anthropometrical index.

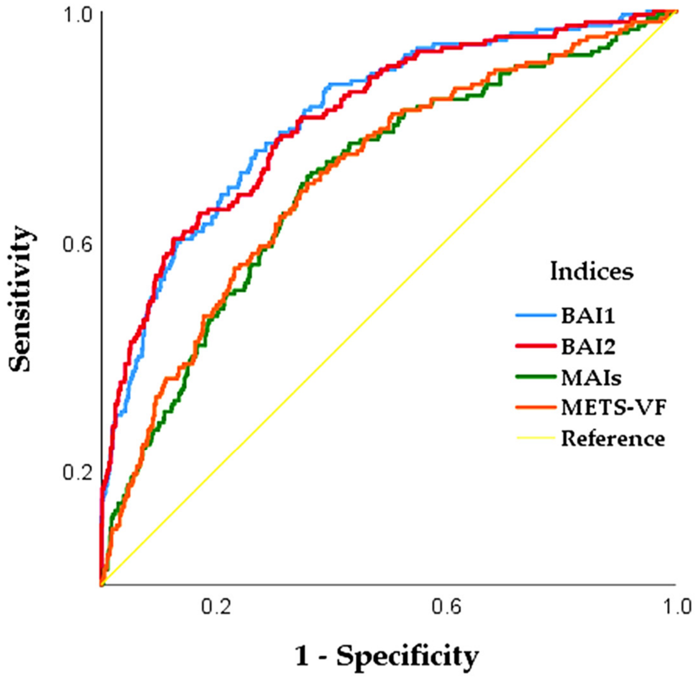

Index	AUC (95% CI)	*p*
BAI1	0.82 (0.78 – 0.85)	<0.001
BAI2	0.81 (0.78 – 0.85)	<0.001
MAIs	0.70 (0.66 – 0.75)	<0.001
METS-VF	0.71 (0.66 – 0.76)	<0.001

Figure S3. ROC curve analysis and AUC comparison of BAIs, MAIs, and METS-VF to identify hypertension in Mexican adults. A *p*-value ≤ 0.05 was taken as statistically significant. Abbreviations: ROC: Receiver operating characteristic, AUC: Area under the curve, CI: Confidence interval and BAI: Biochemical-anthropometrical index, MAIs: Mexican adiposity indices and METS-VF: Metabolic score for visceral fat.
